# Leaf stoichiometry of European beech (*Fagus sylvatica* L.) and pedunculate oak (*Quercus robur* L.) seedlings grown on an innovative peat-free organic substrate

**DOI:** 10.1038/s41598-025-23221-6

**Published:** 2025-11-12

**Authors:** Michał Jasik, Karolina Staszel-Szlachta, Stanisław Małek

**Affiliations:** Department of Ecology and Silviculture, Faculty of Forestry, University of Agriculture in Kraków, Al. 29 Listopada 46, Kraków, 31-425 Poland

**Keywords:** European beech, Pedunculate oak, Peat-free substrate, Leaf stoichiometry, Seedlings nutrition, Ecology, Ecology, Environmental sciences, Plant sciences

## Abstract

The impact of changing climate conditions on the stability, structure and biodiversity of forest ecosystems in Europe is well known. The main threat to trees is the continuous increase in temperature and changes in moisture conditions, especially in the soil. Very often, seedlings with a covered root system grown in container nurseries are used to rebuild forests or replace decaying spruce monocultures. The cultivation of such seedlings is carried out on a substrate whose main component is peat, the extraction of which poses a serious threat to the environment. Leaf nutrient stoichiometry offers an important indicator of the nutritional status of forest tree seedlings. This study assessed the potential of alternative peat-free substrates in nursery production and evaluated European beech and pedunculate oak seedlings with a covered root system, examining whether it ensured their proper nutrition and appropriate stoichiometric ratios of macroelements in the assimilation apparatus when compared to seedlings produced under the same conditions on a peat substrate. These studies were carried out in the production of beech and oak seedlings on innovative, organic, peat-free substrates using standard fertilization and a new fertilization developed by the research team. The peat-free substrates were characterized by higher concentrations of nitrogen (N), potassium (K), and phosphorus (P), which promote more effective growth. Our elemental leaf stoichiometry results indicate their excess in relation to standards, which suggests that plants can use these elements as reserves for the future. Results also showed strong correlations between the contents of elements in the soil and the growth parameters of seedlings. The peat-free substrates showed a beneficial effect on seedling growth, thus highlighting their potential as suitable substrates in nurseries.

## Introduction

 Changing climate conditions are having a profound impact on global photosynthesis and posing a threat to forest ecosystems in Europe, negatively impacting their stability, structure, and biodiversity^1^. Recent research clearly shows that the severity of soil and ecological droughts and changes in moisture conditions have significantly increased in recent years during the growing season, posing a serious threat that directly impacts forest productivity^2^. It is predicted that in the long term, climate change, particularly the severity of droughts, will lead to increased water stress, and the occurrence of extreme weather events may pose some of the most serious threats to forest productivity through decreased biomass growth and increased tree mortality worldwide^3^. Moreover, a steady increase in the frequency of extreme disturbances, such as long-term droughts, floods, and heat waves, is predicted^4^. Global warming will therefore have a significant impact on European forest ecosystems and may result in a shift in the competitiveness and dominance of species with contrasting ecological characteristics and varying drought tolerance, as well as their ability to regenerate quickly. Mixed stands will therefore become an important component^5,6^. Beech (*Fagus sylvatica* L.) is the most widespread forest tree in the temperate zone of Europe, naturally occurring on a wide range of soil types^7^. Its range is expanding northwards and to higher mountain locations in response to the changing climate—an expansion that may cause the species to eventually replace spruce monocultures (*Picea abies* L.)^8^. However, scientists predict changes in the distribution of the species in Europe especially in mountainous regions where rising temperatures and an increase in the frequency of extreme weather events such as drought or water scarcity are leading to a significant reduction in habitats suitable for beech at lower elevations (below 500 m above sea level)^9^.

The second most economically and ecologically valuable tree species in Central Europe is the pedunculate oak (*Quercus robur* L.)^10^. This species is considered one of the most important elements of mixed stands with the ability to adapt to climate change^11^. In stands, oak is underplanted with shade-tolerant tree species that are more competitive in order to obtain high-quality wood^12^. This competition hampers the natural regeneration of the oaks, necessitating their regeneration via artificial methods. In addition, the species could play a key role in the adaptation of Europe’s forests to changing climatic conditions, as they show greater resistance to heat stress and drought compared to the common beech. Models assume that the range could expand northward with the oak’s presence at higher elevations previously overlooked due to colder climates^10,13^.

Very often, seedlings with a covered root system grown in container nurseries are used to rebuild forests or replace decaying spruce monocultures. The cultivation of such seedlings is carried out on a substrate whose main component is peat, the extraction of which poses a serious threat to the environment. Peatlands are known to store significant amounts of soil carbon (estimated at up to one-third of the global supply) and have the potential to mitigate climate change^14,15^. Peatlands occupy a small part of the world’s land surface, but their degradation releases 2 billion metric tons of CO^2^ emissions into the atmosphere^16^. In recent years, in several European countries such as Austria, England, Germany, Finland, Ireland, Northern Ireland, Scotland and Wales, measures are being taken to protect, restore and carry out sustainable management over peatland use depending on the recognized condition of the habitat^17^. Therefore, it is becoming extremely important to conduct research on creating an alternative substrate to peat^18–20^, with composted municipal waste or biocomposts cited among the materials with which to replace peat^21,22^.

Some researchers have focused on dead wood as a substrate that provides favorable physicochemical, biochemical, and moisture properties for seedlings of various tree species^23–26^. The most important consideration in the production of an alternative substrate is to provide appropriate chemical properties for plant growth and the proper nutritional status of the seedling. A well-analyzed component in the literature is coconut fiber, which has a high pH, ​​high potassium content, and a high C: N ratio, which may negatively affect nitrogen availability^27,28^. In turn, the use of green compost is difficult to analyze and evaluate due to its variable chemical properties; another problem resulting from the use of this type of substrate is increased susceptibility to fungal pathogens, weed seeds, or insects, which would adversely affect the development of seedlings^29^. On the other hand, post-logging waste in the form of bark and wood chips has shown high contents of toxic phenolic compounds and other components that can be phytotoxic and constitute a barrier for plants planted on such a substrate^30^. Therefore, it is extremely important to select a substrate that will meet the most important chemical, physical, and biological criteria for seedling growth.

To assess the potential of alternative substrates in nursery production, it is necessary to assess the nutrition of the seedlings grown on them. Leaf stoichiometry, which deals with the balance of multiple elements in ecological interactions and plays an important role in assessing nutrient uptake, plant composition, and ecosystem functions, can be used for this purpose^31–33^. Good growth is conditional on appropriate plant nutrition, and especially on maintaining the appropriate proportions of nutrients in plant tissues^34^. Nutrient concentrations, and their mutual relationships, in the leaves of forest trees are important indicators of the trees’ functioning. The concentration of nutrients in leaves depends on many factors: the amount and availability of water, the length of the vegetation period, the content of nutrients in the substrate, ionic antagonisms, mobility, and ability to absorb ions^35^. Some authors have highlighted the imbalance of proportions between macroelements in European beech leaves, especially the negative trend in phosphorus, calcium, and magnesium nutrition^36,37^. In our study, we evaluated the potential of using an innovative peat-free substrate for the production of beech and oak seedlings with a covered root system compared to the standard peat used, checking whether the new substrate provides proper nutrition to the seedlings and appropriate stoichiometric ratios of macronutrients in the assimilation apparatus. In addition, we analyzed the effect of fertilization (solid and liquid) as a supplement to macronutrients.

## Materials and methods

### Experimental design

**Fig. 1:**
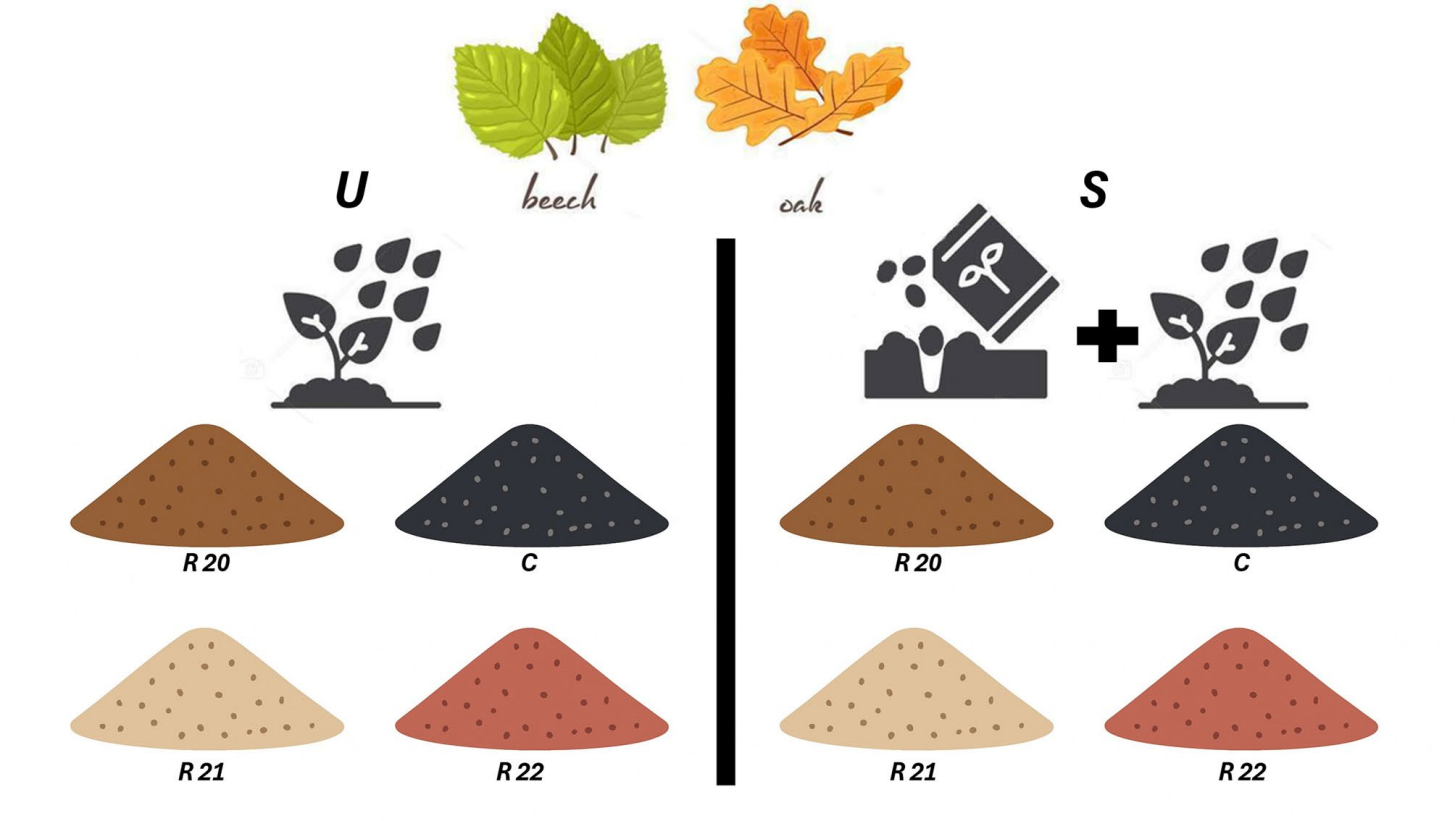
Schematic representation of different types of substrates and fertilizers.

 The experiment was conducted at an open container nursery in Suków (Suków Seed and Nursery Farm in Poland, 50˚ 47′ 46′′ N, 20˚ 42′ 37′′ E) from April to October 2022. Two species of trees, European beech (*Fagus sylvatica* L.) and pedunculate oak (*Quercus robur* L.), were grown in either a standard peat substrate consisted of 93% peat and 7% perlite, supplemented with dolomite at a rate of 3 kg per cubic meter to achieve a pH of 5.5. In the case of peat substrate, the same standard procedure for preparing the substrate for nursery production was used and an innovative peat-free substrate based on natural organic components from agricultural production residues as blends of various organic and inorganic materials, including scobs, wood chips, straw, bark, perlite, core wood, and mixed silage, with proportions expressed in percentages, the pH value of peat-free substrates ranged between 5.3 and 5.8. This substrate was produced in cooperation with Bioefekt Sp. z o.o. Additionally, two methods of seedling fertilization during the growing season were developed: U (for the variant developed at the University of Agriculture in Krakow in which liquid fertilizer was added) and S (for the variant used by the forest nursery in Suków in which Osmocote solid fertilizer was added in this variant, the standard procedure for applying fertilization in forest nurseries was also applied). The variants were labeled as R20, R21, R22-, and C-control (peat), taking into account the fertilization variant; eight variants were analyzed in total (Fig 1). The composition of individual components is presented in Table 1. While detailed information on the chemical and physical properties of the substrate can be found in Rotowa et al.^20^. Beech and oak seedlings were grown in standard 275 cm^3^ Marbet V300 polystyrene containers. The same environmental conditions prevailed in the open container nursery: temperature, humidity, rainfall, irrigation and amount of sunlight. In the production field, 8 columns and 9 rows of cassettes were placed, each with 54 seedlings, two species, two fertilizer variants (solid and liquid), 3 peat-free substrates and cotrola (peat), which in total gave nearly 62,208 seedlings). Substrate and plant samples were collected in July in the middle of the growing season to determine the appropriate nutritional status of the seedlings. The other seedlings grew until the end of the growing season and then overwintered in special cold storage halls to be planted on the crop in the forest.

**Table 1:** Composition of individual components of organic substrates.

Substrate variants	Scobs	Wood chips	Straw	Wood bark	Perlite	Core wood	Mixed silage
	[%]						
R20	73	10	–	10	4	2	1
R21	20	63	–	10	4	2	1
R22	50	–	10	33	4	2	1

### Preparation of material and conduct of the experiment

After filling the containers with different substrate variants, beech and oak seeds were sown by hand at the Sukow-Papierna Nursery Farm (Daleszyce Forest District, Poland) on April 19–20, 2022, according to the standard seed sowing procedure. For this purpose, oak seeds were subjected to scarification involving the removal of about one-third of their cotyledon part immediately before sowing. Beech seeds were stratified under refrigeration at + 3 °C and 31% relative humidity). After sowing, the containers were placed in a vegetation hall for four weeks before being moved to an outdoor production field.

During the period of seedling growth, the rainfall total was only 78 mm, so irrigation was applied using an automatic sprinkler frame from RATHMAKERS Gartenbautechnik to make up for the water shortage. Osmocote fertilizer was applied once during substrate preparation before sowing at a total rate of 3 kg/m³ of each soil, prepared as a mixture of Osmocote 3–4 M (2 kg) and Osmocote 5–6 M (1 kg) in the S variant. The composition of Osmocote 3–4 M fertilizer was as follows: *N* − 16%, including 7.1% N-NO₃ - and 8.9% N-NH₃ +; P₂O₃ − 9%, K₂O − 12%; MgO − 2.0% and micronutrients (B, Fe, Cu, Mn, Zn, Mo); 5–6 M: *N* − 15%; including 6.6% N-NO₃ - and 8.4% N-NH₃ P₂O₃ − 9.0%; K₂O − 12%; MgO − 2.0%; and micronutrients (B, Fe, Cu, Mn, Zn, Mo). The new liquid fertilizer (U variants) was applied based on two different compositions. The first variant consisted of N 4.78%, P₂O₃ 1%, K₂O 2.64%, CaO 2.65%, MgO 1.4%, SO₃ 0.71% and Na₂O 0.14%. This fertilizer was initially applied in a total volume of 3.14 dm^3^ (0.048 dm^3^ 1 m^−2^). The second fertilizer variant contained 0.798% N, 0.166% P₂O₂, 0.440% K₂O, 0.441% CaO, 0.234% MgO, 0.118% SO₂ and 0.023% Na₂O. The second fertilizer was applied in a total volume of 15.09 dm^3^ (0.229 dm^3^ 1 m^−2^). During seedling production, the first fertilizer variant was applied eight times at 10-day intervals, while the second variant was applied fifteen times at 5-day intervals. It should be noted that fertilization patterns remained consistent for both beech and oak seedlings.

**Fig. 2:**
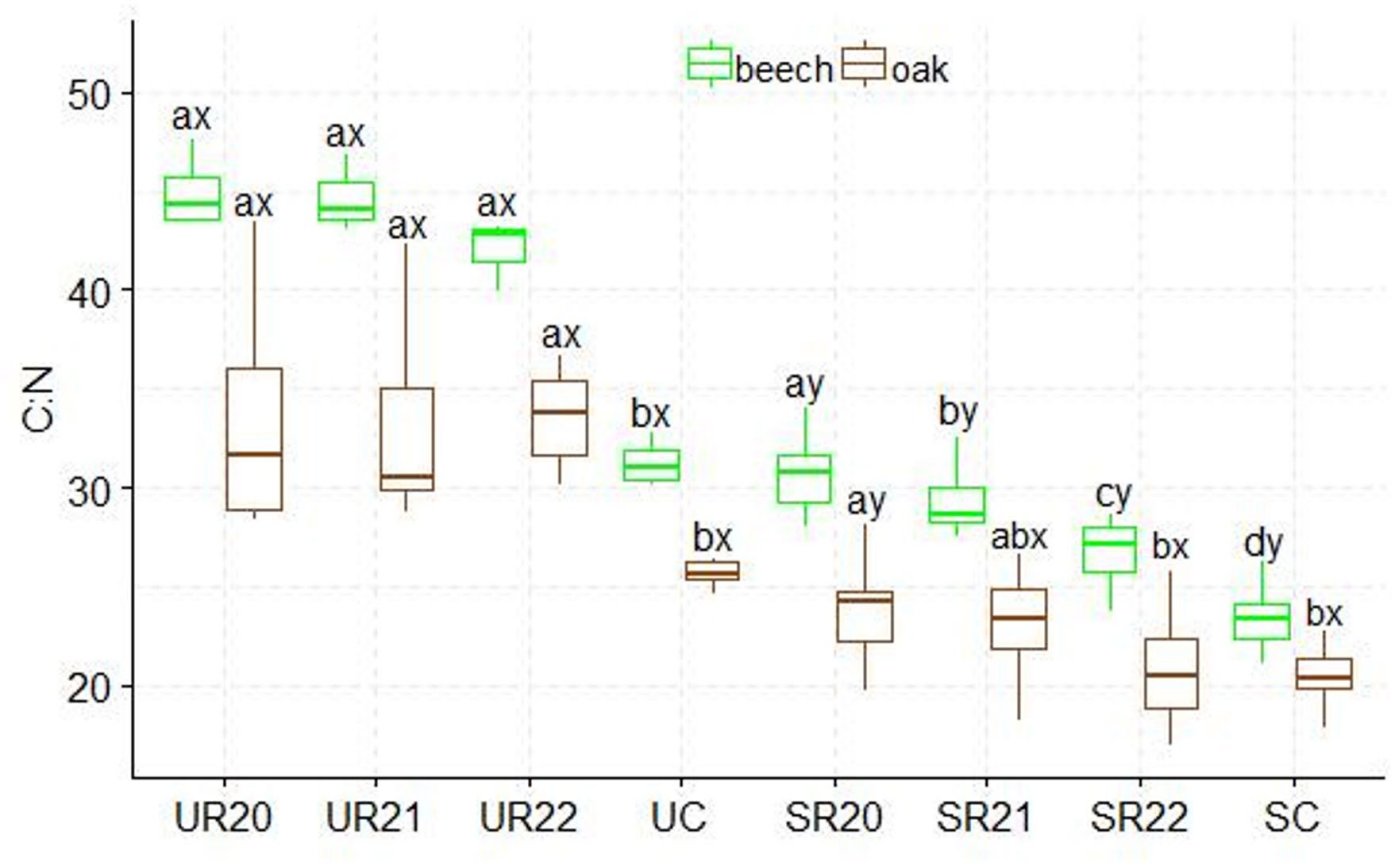
C:N stoichiometry in beech and oak leaves depending on the substrate and production method abc- difference between different substrates, xy- difference between fertilization method; Tukey *HSD p* < 0.05; (box-whisker plots with median, 25- and 75-percentiles).

### Chemical analysis

Fifteen fresh seedlings were randomly selected for each fertilization, substrate, and species (240 seedlings in total), cleaned, and measured (height and diameter at the root collar). Then the leaves, root system, and shoot of each seedling were separated, dried at 65 °C for 48 h, and weighed. In the leaf samples, the concentrations of P, K, Ca, and Mg were determined using an inductively coupled plasma optical emission spectrometer (ICP-OES; Thermo iCAP 6500 DUO, Thermo Fisher Scientific, Cambridge, U.K.). Dried samples of leaves were mineralized in a 3:1 mixture of HNO_3_ and HClO_4_. Carbon (C) and nitrogen (N) in the leaf samples were measured with an elemental analyzer (LECO CNS TruMac Analyzer, Leco, St. Joseph, MI, USA). During operation of the ICP-OES device used in the chemical analysis, the radio frequency (RF) power settings were 1050 W, the plasma gas flow rate was 11 L/min, and the auxiliary gas flow rate was 0.6 L/min. Additionally, 10 soil samples collected near the seedling were prepared and transported to the laboratory, where they were dried at 65 °C for 48 h. After drying, the samples were ground, prepared into approximately 0.5 g samples, and then mineralized in a 3:1 HNO_3_ and HClO_4_ mixture and analyzed by ICP-OES. The soil samples were analyzed for P, K, Ca, and Mg content. Total nitrogen (N) content was determined by dry mineralization in a stream of oxygen with IR detection using a LECO CNS 2000 instrument. The pH in H_2_O and EC of the collected soil samples was determined potentiometrically using an Elmetron CX-705 multiparameter meter.

### Statistical analysis

Non-parametric Spearman’s rank correlation coefficient was used to determine the strength and significance of relationships between inter-variable stoichiometry and the content of individual elements in the soil and leaves of seedlings, as well as basic biometric parameters of seedlings, using the cor.test() function from the base stats package. Relationships between variables were modeled with Generalized Linear Models (GLM) using the glm() function. Depending on the distribution of the data, an appropriate model was selected taking into account the substrate variant and fertilization. For post-hoc tests (multiple comparison analysis) after the significance of effects in GLM models, the emmeans (Estimated Marginal Means) package was used, using the emmeans() function and pairs() for pairwise comparisons using the Tukey method. Results were considered statistically significant at α < 0.05. All statistical analyses were carried out using R statistical software (R Core Team 2022), R Studio (RStudio Team 2022), and Statistica 13 software (2017). Statistical analysis was performed on the results obtained from 15 leaf samples (15*8 = 120) and 10 substrate samples (10*8 = 80) for each variant.

## Results

### Chemical analysis of different substrate variants

The chemical properties of the different organic substrates influenced by oak varied markedly from those of the peat substrate commonly used in the nursery.Variants with solid fertilization (SC) showed pH in the range of 5.35–5.72 and did not differ significantly from each other, except for SR22 (5.41), which showed a statistically lower pH than SR21 and SR20. In the case of electrolytic conductivity (EC), differences between variants and fertilization were much more pronounced. The lowest EC values were recorded in the UR and UC control groups (136–190 µS cm⁻¹), while variants with Osmocote fertilizer significantly increased EC, especially SR21 (1314 µS cm⁻¹), SR22 (1145 µS cm⁻¹), and SC (1013 µS cm⁻¹). The highest N content was found in variants UR21 and SR21, and the lowest in variants UR22 and SR20. Statistically significant differences in Ca content were found between all substrates; the highest values were found for UC variant, and the lowest for variants UR 20 and SR20. Different relationships were also found in the case of K content, in which the lowest values were found in variants UR20 and UR21, while the highest were found in UR22 variant. A similar trend was observed in the case of P. For K, Mg, and P content, statistically significant differences were found between the UC and SC production methods. Twice-as-high Mg values were found in the UC variants compared to SC. Chemical analysis of the substrates showed that some parameters were statistically significantly higher in oak than in beech seedlings in particular in variants with liquid fertilization in N, Ca K and Mg and with the use of solid fertilization in P (Table 2). In the case of beech, soil pH was slightly higher than in the oak soil and showed greater variation between the treatments. The highest pH values were found in the UR20 group, while the lowest were found in the UC treatments. A significantly lower pH was observed in the control treatments and in R22 compared to the other treatments. As with oak, electrolytic conductivity (EC) significantly increased after the application of solid fertilizers. The lowest EC was found in the UR20 treatment groups, while the highest were found in the SC treatment. The substrates on which beech seedlings were grown differed significantly between the UC and SC production method (liquid and solid) in terms of the content of individual elements, except for Ca. The highest N content was found in the SC control variants, while the lowest was found in the UR20. Similar relationships were found for the Ca and Mg content: The contents of these elements in the SC variant were statistically significantly different from that of the other substrates. The contents of K and P were highest in the UR22 and SR22 variants (Table 3).

**Table 2:** Nutrient content of the substrate in oak containers.

Fertilizer	pH	EC	*N*	Ca	K	Mg	*P*
UR20	5.91 ± 0.17^ax^	165 ± 41^aby^	0.68 ± 0.02^bx^*	0.61 ± 0.07^cx^	0.07 ± 0.01^by^*	0.06 ± 0.01^by^*	0.03 ± 0.00^dy^
UR21	5.71 ± 0.08^bx^	154 ± 19^by^	0.77 ± 0.03^ay^*	0.68 ± 0.03^cy^*	0.07 ± 0.01^by^*	0.05 ± 0.00^by^	0.04 ± 0.00^cy^*
UR22	5.80 ± 0.08^abx^	190 ± 23^ay^	0.62 ± 0.02^cy^*	1.02 ± 0.11^by^*	0.12 ± 0.03^ay^	0.06 ± 0.01^by^*	0.05 ± 0.00^by^
UC	5.31 ± 0.15^cx^	136 ± 16^by^	0.76 ± 0.03^ax^*	1.82 ± 0.52^ax^	0.09 ± 0.03^aby^*	0.88 ± 0.17^ax^*	0.02 ± 0.00^ay^*
SR20	5.71 ± 0.14^ay^	525 ± 166^bx^	0.62 ± 0.06^cy^	0.60 ± 0.13^cx^*	0.14 ± 0.04^bx^	0.08 ± 0.02^cx^	0.09 ± 0.01^cx^*
SR21	5.72 ± 0.11^ax^	1314 ± 335^ax^	1.13 ± 0.11^ax^*	0.92 ± 0.09^bx^*	0.31 ± 0.05^ax^*	0.12 ± 0.02^bx^*	0.22 ± 0.03^ax^*
SR22	5.41 ± 0.19^by^	1145 ± 325^ax^	0.93 ± 0.11^bx^	1.22 ± 0.06^ax^	0.27 ± 0.07^ax^	0.10 ± 0.02^bcx^	0.15 ± 0.02^bx^*
SC	5.35 ± 0.25^bx^	1013 ± 144^ax^	0.70 ± 0.06^cy^*	1.30 ± 0.16^ay^*	0.26 ± 0.04^ax^*	0.45 ± 0.04^ay^	0.11 ± 0.02^cx^

mean ± SD; N, P, K, Ca, Mg [%]; small letters abc- difference between different substrates, xy- difference between fertlization method * difference between beech; Tukey HSD *p* < 0.05.

**Table 3:** Nutrient content of the substrate in beech containers.

Feltilizer	pH	EC	*N*	Ca	K	Mg	*P*
UR20	6.03 ± 0.08^ax^	109 ± 11^by^	0.43 ± 0.01^dy^	0.62 ± 0.04^cy^	0.03 ± 0.01^ay^	0.05 ± 0.01^by^	0.03 ± 0.00^by^
UR21	5.93 ± 0.06^bx^	161 ± 27^ay^	0.55 ± 0.02^cy^	0.74 ± 0.05^cx^	0.06 ± 0.01^ay^	0.05 ± 0.01^by^	0.04 ± 0.00^ay^
UR22	5.73 ± 0.05^cy^	177 ± 58^ay^	0.56 ± 0.01^by^	1.37 ± 0.33^bx^	0.09 ± 0.12^ay^	0.07 ± 0.01^by^	0.04 ± 0.00^ay^
UC	5.49 ± 0.08^dx^	173 ± 26^ay^	0.72 ± 0.01^ay^	1.69 ± 0.18^ax^	0.05 ± 0.00^ay^	0.61 ± 0.09^ax^	0.02 ± 0.00^cy^
SR20	5.80 ± 0.07^ay^	609 ± 165^bx^	0.67 ± 0.07^bx^	0.73 ± 0.04^cx^	0.13 ± 0.03^bx^	0.10 ± 0.01^bx^	0.12 ± 0.02^abx^
SR21	5.87 ± 0.08^ax^	997 ± 225^ax^	1.00 ± 0.09^ax^	0.75 ± 0.07^cx^	0.23 ± 0.07^ax^	0.10 ± 0.01^bx^	0.14 ± 0.02^ax^
SR22	5.52 ± 0.13^bx^	1039 ± 369^ax^	0.96 ± 0.10^ax^	1.24 ± 0.09^bx^	0.26 ± 0.07^ax^	0.11 ± 0.02^bx^	0.11 ± 0.02^bx^
SC	5.44 ± 0.1^bx^	1135 ± 272^ax^	1.03 ± 0.13^ax^	1.52 ± 0.07^ay^	0.25 ± 0.09^ax^	0.51 ± 0.04^ay^	0.10 ± 0.02^bx^

mean ± SD; N, P, K, Ca, Mg [%]; small letters abc- difference between different substrates, xy- difference between fertilization method * difference between beech; Tukey HSD *p* < 0.05.

### Stoichiometry of elements in leaves

**Fig. 3:**
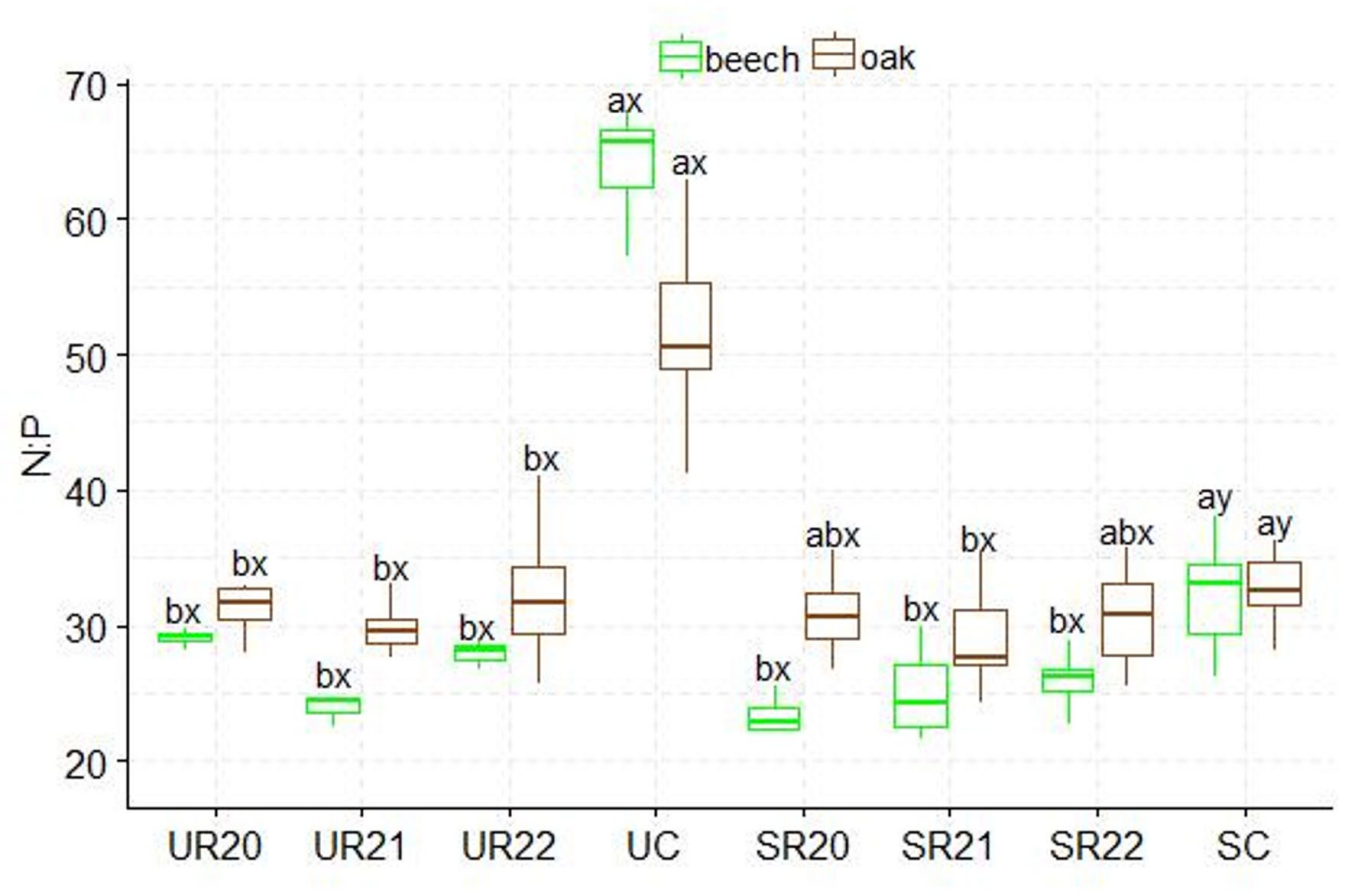
N:P stoichiometry in beech and oak leaves depending on the substrate and production method abc- difference between different substrates, xy- difference between fertilizationn method; Tukey *HSD p* < 0.05.

**Fig. 4:**
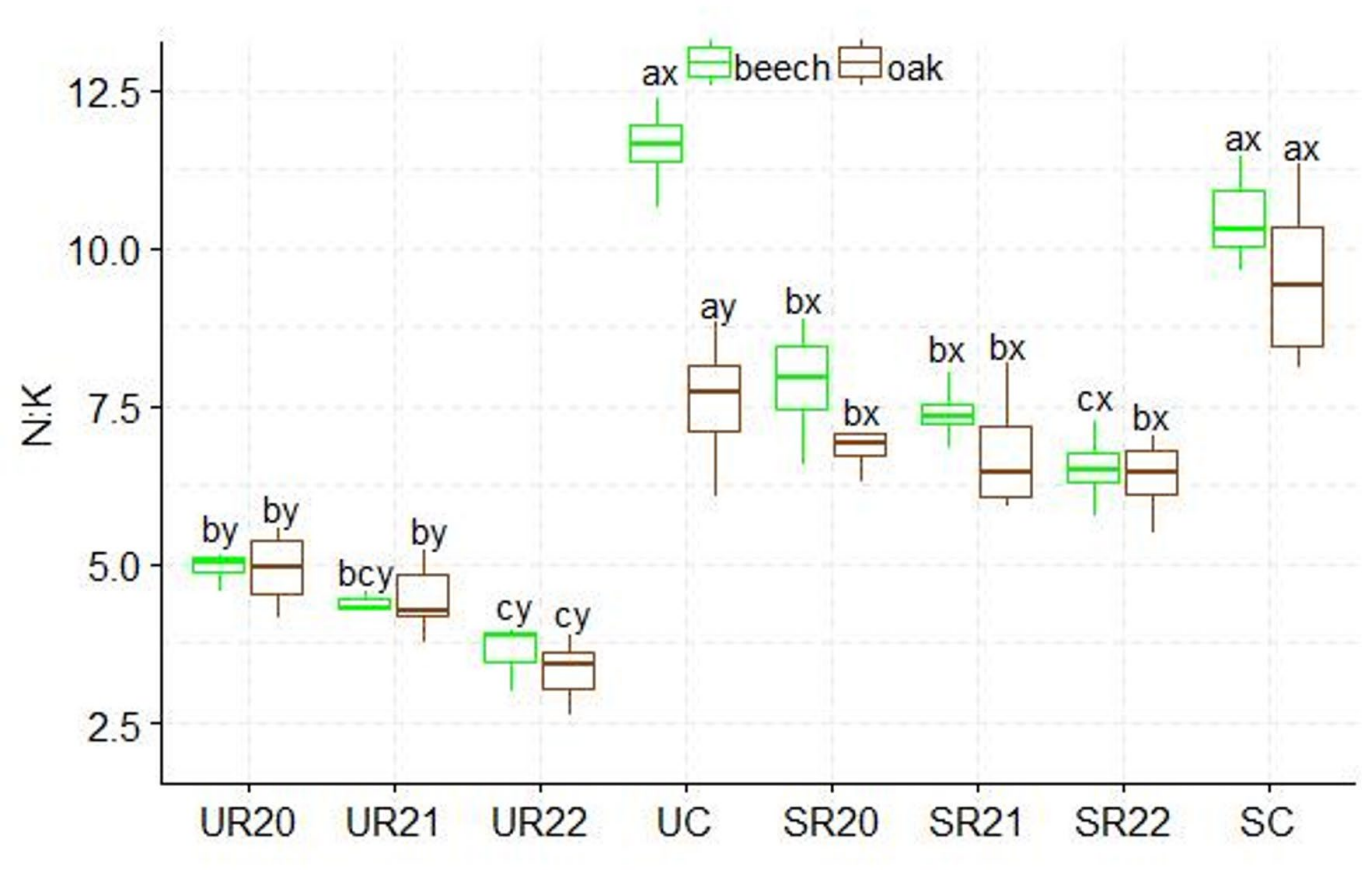
N:K stoichiometry in beech and oak leaves depending on the substrate and production method abc- difference between different substrates, xy- difference between fertilization method; Tukey *HSD p* < 0.05.

**Fig. 5:**
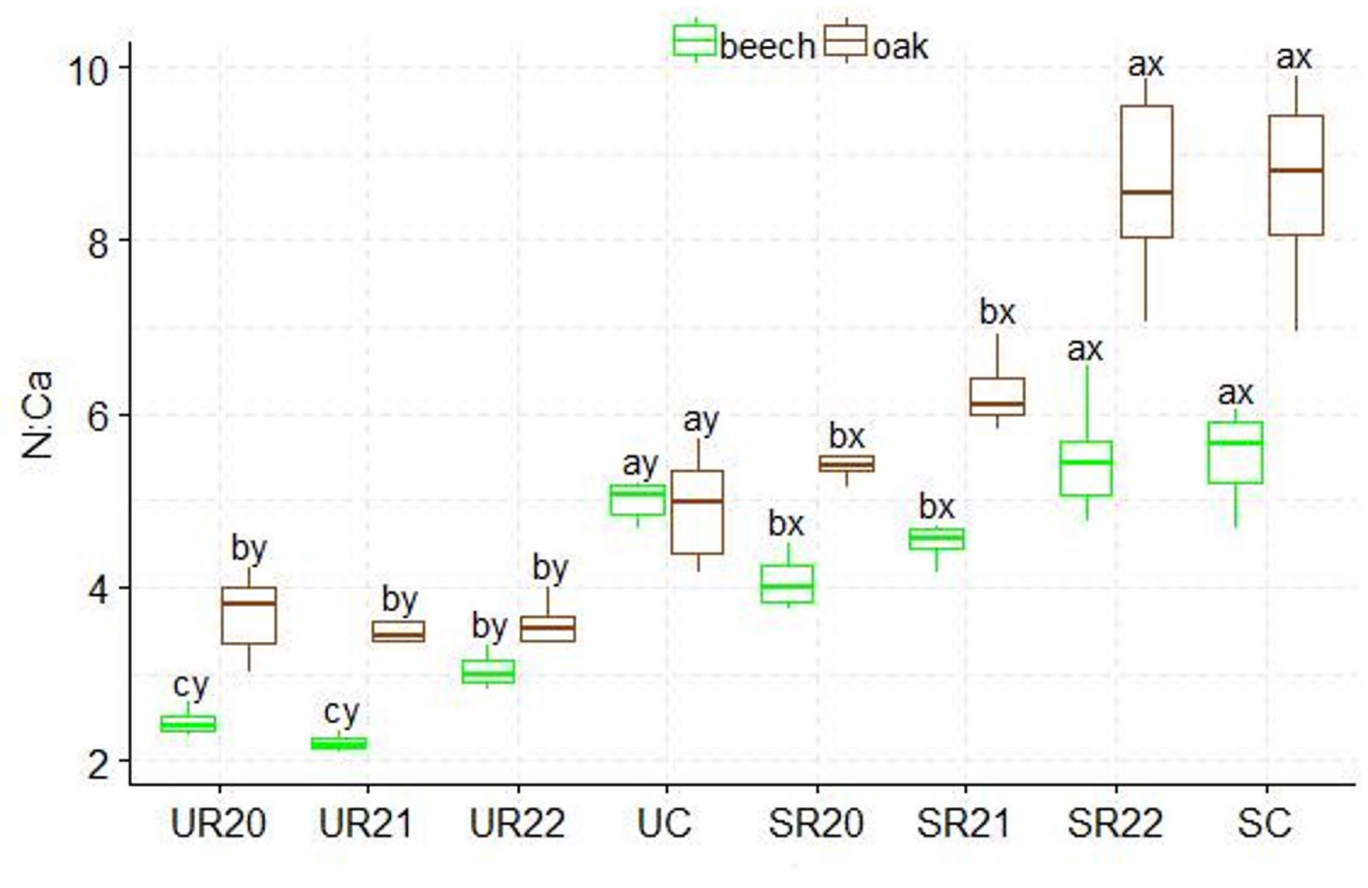
N:Ca stoichiometry in beech and oak leaves depending on the substrate and production method abc- difference between different substrates, xy- difference between fertilization method; Tukey *HSD p* < 0.05.

**Fig. 6:**
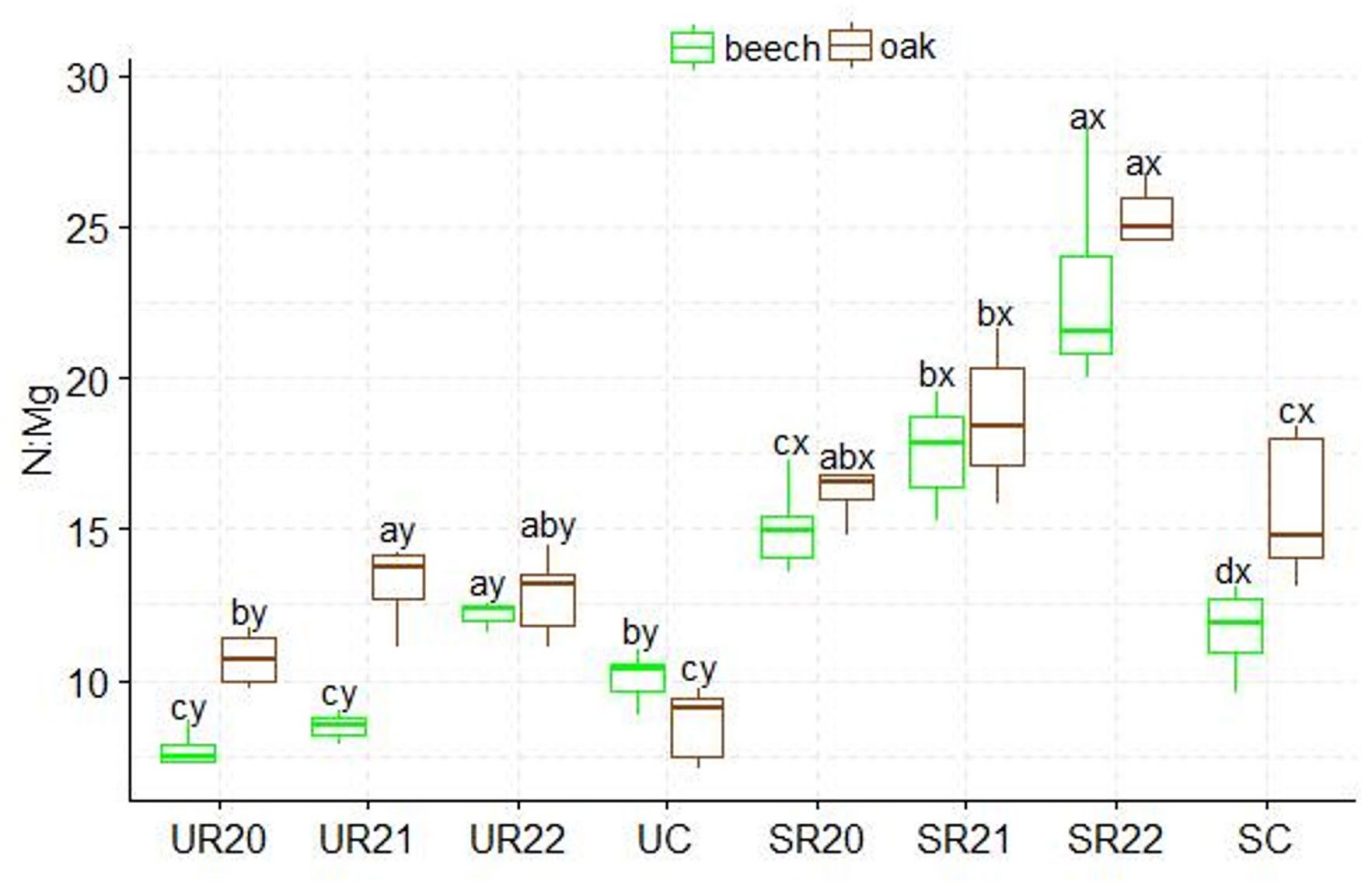
N:Mg stoichiometry in beech and oak leaves depending on the substrate and production method abc- difference between different substrates, xy- difference between fertlization method; Tukey *HSD p* < 0.05.

A higher C: N ratio was found in the substrates influenced by beech seedlings than in those influenced by oak. The UR substrate variants differed from the SC control variant. The highest C: N values were found in the SR20 variant, and the lowest in the SC control variant. The SR substrates influenced by both species showed statistically significant differences in C: N ratio (Figs.  2). The highest N: P ratio was found in the UC control variant, which differed significantly from the other UR substrates influenced by both species. Statistically significant differences in production method were only found between the UC and SC control variants. In beech, the SC control variant differed significantly from the other variants in N: P ratio, and in oak, N:P differences were found between the SC control variant and SR21 (Fig. 3). Significantly higher N: K ratios were found in the UC and SC control variants; in addition, statistically significant differences in N: K ratios were found between the fertilization method and the substrate. The lowest N: K values were found in the UR and SR22 variants for both seedling species (Fig. 4). The control variant UC, SC, and SR22 showed the highest N: Ca ratios in oak and beech, and differed from the other substrate variants. Statistical differences in N: Ca ratios were found in the production methods of UC and SC (Fig. 5). SR22 differed significantly from the other substrates in terms of the N: Mg ratio, and the lowest N: Mg values were recorded in the control variant SC. In the case of UC fertilization, the lowest N: Mg values were recorded for UR20 and the highest in UR22 in both species (Fig. 6).

### Analysis of biometric parameters in relation to chemical parameters

**Fig. 7:**
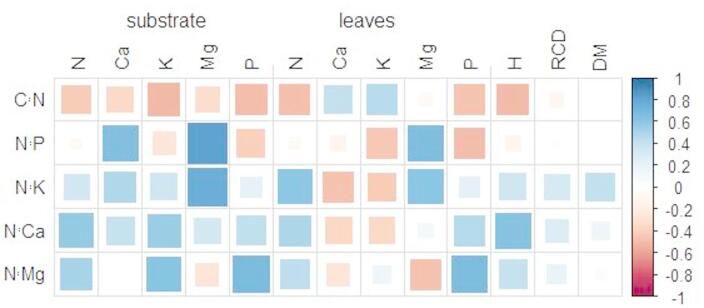
Correlation between stoichiometry and chemical properties in substrate and leaves (H-height [cm], RCD- root collar diameter [mm], DM-dry mass [g]).

Analysis of biometric parameters revealed a significant effect of the fertilization variant and substrate used on the growth of both oak and beech, with plant response differing depending on the species and growth parameter. In the case of oak, the highest mean height (H) was obtained in the S variants (SR22: 25.41 cm; SC: 24.15 cm), which was significantly higher compared to the R variants and the UC control (ca. 12.70–13.77 cm). UR fertilizers did not differ significantly in their effect on height. Seedling diameter (D) did not show significant differences between the variants; all values ranged from 3.76 to 4.24 mm. Similarly. Dry matter (DM) in the case of oak did not differ significantly between fertilizers – values ranged from 3 to 5 g. Beech, on the other hand, showed a stronger response to the applied fertilization than oak. The highest average height was achieved in the SC variant (38.35 cm), which was significantly higher than in all other variants. SR variants (SR20–SR22) also contributed to a significant increase in height (17.15–24.20 cm), compared to UR fertilizers and the UC control (9.55–13.12 cm). Shoot diameter (D) also increased under the influence of fertilizer application. The highest diameter was obtained in the SC variant (4.60 mm), while the lowest values were recorded in the UR20 group (2.60 mm). The use of the SR and SC variants significantly increased the dry matter (DM) of beech. The highest values were obtained in the SC group (4.56 g), as well as in SR22 (2.79 g) and SR21 (2.30 g). These values were significantly higher than in the UR and UC variants, where the dry matter did not exceed 1.20 g (Table 4). The correlation showed a strong positive relationship between the stoichiometry of N:P and N:K and that of Ca and Mg, with the ratio of N:Ca to N and K and N:Mg to N, K, and P. A negative relationship of C:N was shown between the analyzed elements in the substrate (N, P, K, Ca, Mg). Elements in leaves, such as N and P, showed a negative relationship with C:N, while a positive effect was found with N:K, N:Ca, and N:Mg. Mg showed a stronger positive correlation with N:P and N:K. The content of Ca and K in leaves showed a positive relationship, while the remaining stoichiometric ratios showed a negative relationship. Higher N: Ca, N:K, and N: Mg ratios had a positive effect on the height of the analyzed seedlings. The remaining parameters of the seedling root-collar diameter and diameter showed a positive effect of N:K (Fig. 7). GLM analysis confirmed the significant influence of species, individual elements, and fertilization on the substrate and nutritional status of the seedlings, as expressed by the contents of individual elements in the leaves (Table 5).

**Table 4:** Biometric parameters of beech and oak seedlings depending on the substrate and fertilization.

Fertilizer	Oak			Beech		
	H	D	DM	H	D	DM
UR20	13.77 ± 2.46^ax^	4.17 ± 0.65^ax^	4.23 ± 1.09^ax^	9.55 ± 1.21^by^	2.60 ± 0.26^ay^	0.81 ± 0.23^ay^
UR21	13.39 ± 5.05^ax^	3.76 ± 0.66^ax^	3.51 ± 1.39^ax^	10.89 ± 2.02^aby^	2.66 ± 0.25^ay^	0.92 ± 0.29^ay^
UR22	13.03 ± 3.57^ay^	3.90 ± 0.64^ax^	3.55 ± 0.39^ax^	10.49 ± 2.13^aby^	2.75 ± 0.410^ay^	0.97 ± 0.28^ay^
UC	12.70 ± 3.71^ay^	4.20 ± 0.63^ax^	4.48 ± 1.35^ax^	13.12 ± 3.99^ay^	2.83 ± 0.58^ay^	1.20 ± 0.48^ay^
SR20	13.30 ± 4.59^bx^	3.77 ± 0.76^ax^	3.06 ± 1.30^ay^	17.15 ± 5.45^bx^	3.51 ± 0.75^bx^	1.65 ± 0.70^cx^
SR21	19.38 ± 8.82^abx^	4.14 ± 0.56^ax^	4.04 ± 1.20^ax^	21.93 ± 4.33^bx^	3.80 ± 0.47^abx^	2.30 ± 0.51^bcx^
SR22	25.41 ± 10.36^ax^	4.24 ± 1.03^ax^	4.52 ± 2.36^ax^	24.20 ± 6.22^bx^	4.13 ± 1.05^abx^	2.79 ± 0.97^bx^
SC	24.15 ± 9.32^ax^	4.03 ± 1.22^ax^	5.20 ± 3.38^ax^	38.35 ± 7.79^ax^	4.60 ± 0.63^ax^	4.56 ± 1.39^ax^

mean ± SD; H- height cm; D- diameter mm; DM- dry mass g; small letters abc- difference between different substrates, xy- difference between production method; Tukey HSD *p* < 0.05.

**Table 5:** Summary of GLM analysis for soil macronutrients and leaves.

	Substrate		Leaves	
	F	*p*	F	*p*
Species	25.514	*0.000*	70.82	*0.000*
Nutri	171.960	*0.000*	185.39	*0.000*
Fertilizer	352.780	*0.000*	60.89	*0.000*
Species*Nutri	13.570	*0.000*	4.49	*0.001*

Italic represents statistically significant effect of chemical properties between fertilizer variant, species and substrate; *p* < 0.05.

## Discussion

Chemical analyses of the components used showed significant differences between the peat substrates and the peat-free variants. The increase in conductivity indicates a significantly greater amount of available ions (e.g. nutrients) in the soil after the application of solid fertilizers in the SR variants, which may translate into better plant nutrition and the absorption of elements from the soil. Our research showed that peat-free substrates had quite similar N, K and P contents compared to peat substrates. The higher Ca and Mg contents recorded in peat samples could have resulted from the fact that the substrate was enriched with dolomite. Studies have shown that this additive improves the efficiency of the seedling through its growth and root-system development. Additionally, higher Mg content can increase the success of seedlings by up to 8.5%^38,39^. The peat-free substrate produced had a beneficial effect on the development of root systems due to its physical properties; it had a lower density, which would have affected its granulometric composition and enhanced the transport of nutrients^20,40^. In conditions of nutrient deficiency, seedlings grow toward the nutrient source—which comprises not only the elements within the substrate itself, but also the substances applied via foliar or soil fertilization—faster. Therefore, at the nursery production stage, it is extremely important to provide optimal conditions for seedling growth in the form of an appropriate substrate and fertilization^41^.

The stoichiometric ratios of C, N and P in leaves play a key role in the proper growth of seedlings and in maintaining the balance of biogeochemical processes occurring in forest ecosystems.

In addition, stoichiometry is useful for understanding the efficient use of nutrients by plants and their relative limitation in the forest ecosystem depending on environmental conditions^42^. In the studies of Ognejenović et al.^43^ and in our studies, it was shown that the stoichiometric ratios of elements in leaves mostly exceeded the proposed standards. There is an assumption that an excess of the analyzed elements can be used as a reserve for future growth or constitutes a defense mechanism of the plant (Ågren et al. 2008). In the case of the N: Ca and N: Mg ratios we recorded in beech and oak leaves growing in substrates influenced by foliar fertilization, the values were within the proposed standards, while the SR22 substrate exceeded the standards for both ratios. On this basis, it can be concluded that the analyzed substrates fulfilled one of their most important roles, which is to ensure the proper nutrition of seedlings. The stoichiometry of the most important elements, such as C, N, and P, is determined, among other means, by the balance of nutrient uptake relative to seedling growth, and may depend on light, temperature, and even the amount of released carbon dioxide^44^. An increase in the concentration of carbon dioxide in the atmosphere causes a slower decrease in plant P than in N^45^. In the present study, a higher N: P ratio was noted in oak compared to beech, with values oscillating between 25 and 35, which is comparable to the results of other studies^45,46^. Additionally, a previous study found that an increase in N may lead to a decrease in the content of other elements, such as P, K, Ca, and Mg^47^.

In the present study, a strong negative correlation was observed between the C:N ratio in leaves and the substrate type, and a strong positive relationship between Mg in the substrate and the N:P and N:K content in the leaves of seedlings. In a study by Braun et al.^48^, a negative effect of P concentration in leaves and N:P in the soil was noted, similar to the relationship noted in our experiment. Moreover, depending on the analyzed substrate, a decrease in the concentrations of Ca and Mg was previously noted in the leaves of European beech, and Mg deficiencies could occur in places where the critical value of N in the soil was exceeded^37,48,49^. The lower Ca content in the soil was reflected in the N:Ca stoichiometry in the leaves of both analyzed species. Peat-free UR substrates were characterized by the lowest N:Ca values compared to substrates with the addition of soil fertilization, which had a significant impact on the nutritional status of seedlings and even their growth. This relationship was also noticed by Binkley and Högberg^50^, who observed the response of forests to limitations in the uptake of some elements and the growth response to fertilization, especially with regard to the content of P, the demand of which often exceeds its supply.

The stoichiometry of N:Mg in the UC and SC control substrates is lower compared to the other variants. This relationship may be due to the higher Mg content in the peat substrate, which affected the overall stoichiometry. The negative correlation of C:N and N: P with growth parameters indicates a significant effect of C, N, and P on plant growth, which has been observed in European forests^36,51^. In addition, GLM analysis of elemental content in leaves and soil showed a close relationship between species, fertilization and elemental content in soil, which has a direct bearing on the nutritional status of seedlings and stoichiometry.

Leaf stoichiometry, for both beech and oak, exceeded the limits for beech and oak stands^52^. The ratios of nitrogen to other elements are closer to those obtained in stands highly influenced by nitrogen deposition, due to the widespread use of nitrogen-based fertilizers in nurseries. The stoichiometric limits for N:P, N:K, and N:Ca ratios were exceeded to a greater extent for the peat-based control substrate, which indicates that the new peat-free substrate has a more favorable effect on leaf stoichiometry for both beech and oak. Magnesium-containing dolomite is added to the peat substrate, resulting in a lower N: Mg ratio when using this substrate. Based on the study, it can be concluded that, the proposed alternative substrate is sufficient to provide seedlings with optimal growth and their nutritional status at the nursery production stage.

## Conclusion

Our chemical analyses show that peat and peat-free substrates differ in nutrient contents, which is important for seedling growth. The peat-free substrates were characterized by higher concentrations of nitrogen (N), potassium (K), and phosphorus (P), which promote more effective growth. Adding dolomite to peat increases the content of calcium (Ca) and magnesium (Mg), which also has a positive effect on seedling growth. Our elemental leaf stoichiometry results indicate their excess in relation to standards, which suggests that plants can use these elements as reserves for the future. The N:Ca and N:Mg values in leaves growing in foliar-fertilized substrates were within the standards, confirming that these seedlings’ nutritional status was acceptable. Our results also showed strong correlations between the contents of elements in the soil and the growth parameters of seedlings. The peat-free substrates, despite their lower N:Ca content, showed a beneficial effect on seedling growth, thus highlighting their potential as suitable substrates in nurseries.

## Data Availability

No datasets were generated or analysed during the current study.
